# Association of the Cardioankle Vascular Index and Ankle-Brachial Index with Carotid Artery Intima Media Thickness in Hemodialysis Patients

**DOI:** 10.1155/2013/401525

**Published:** 2013-06-24

**Authors:** Tomohito Gohda, Hiromichi Gotoh, Yoshikazu Gotoh, Saori Yamaguchi, Yasuhiko Tomino

**Affiliations:** ^1^Division of Nephrology, Department of Internal Medicine, Juntendo University Faculty of Medicine, 2-1-1 Hongo, Bunkyo-ku, Tokyo 113-8421, Japan; ^2^Saiyu Soka Hospital and Saiyu Clinic, Saitama, Japan

## Abstract

The objectives of the present study are (1) to compare the cardioankle vascular index (CAVI), ankle-brachial index (ABI), and carotid artery intima-media thickness (CA-IMT) between HD patients with and without type 2 diabetes (T2D) or prevalence of cardiovascular (CV) disease and (2) also to evaluate the relationship of these indices with CA-IMT in these patients according to ABI levels. This study consisted of 132 HD patients with T2D and the same number of patients without T2D. The patients with diabetes or prevalence of CV disease had significantly higher CA-IMT and lower ABI values than those without diabetes or prevalence of CV disease, respectively. Although diabetic patients had higher CAVI than those without diabetes, CAVI did not differ between patients with or without prevalence of CV disease. In univariate analysis, CA-IMT was more strongly correlated with ABI than CAVI. However, the opposite was true in patients with an ABI value of more than 0.95. Both indices were significantly correlated with CA-IMT although ABI was a powerful determinant than CAVI. It appears that both indices are associated with CA-IMT in HD patients, especially with an ABI value of more than 0.95.

## 1. Introduction

Cardiovascular (CV) diseases are major causes of death in patients with end-stage kidney disease (ESKD), especially for patients with type 2 diabetes (T2D). Carotid artery IMT (CA-IMT) is one of the most established predictors of death from CV disease independent of other classical risk factors in hemodialysis (HD) patients [1–3], although recent studies reported that the association between CA-IMT progression assessed from two ultrasound scans and CV disease remains unproven in general population [4, 5].

The ankle-brachial index (ABI) is used to diagnose peripheral artery occlusive disease (PAOD), and for patients, with an ABI value of less than 0.90, it is accepted as a reliable marker for PAOD [6]. A lower ABI value has also been shown to be significantly associated with CV diseases [7]. On the other hand, brachial-ankle pulse wave velocity (baPWV) is a useful marker for measuring arterial stiffness, one aspect of arteriosclerosis [8]. Several studies have demonstrated that both indices reflect the severity of carotid arteriosclerosis and predict all-cause and CV mortality in HD patients [9–12]. However, one drawback of baPWV is that it is affected by changes in blood pressure during measurements. Recently, a novel arterial stiffness parameter, the cardio-ankle vascular index (CAVI), was developed by measuring baPWV and blood pressure. Unlike baPWV, CAVI is independent of blood pressure, and has adequate reproducibility for clinical practice [13]. However, it is also considered to be inaccurate if the ABI value is less than 0.95, as is also the case for baPWV [14].

The objectives of the present study are (1) to compare the CAVI, ABI and CA-IMT between HD patients with and without T2D or history of CV disease and (2) also to evaluate the relationship of these indices with CA-IMT as a surrogate maker of carotid arteriosclerosis in HD patients according to ABI levels since a considerable number of HD patients have low ABI.

## 2. Methods

### 2.1. Patients

Two hundred and sixty-four HD patients, consisting of 132 patients with T2D and the same number of patients without T2D, at the Saiyu Soka Hospital (Soka, Saitama, Japan) were enrolled in the present study. Patients were eligible for enrolment if they had been on HD for at least 6 months. The study protocol was approved by our ethics committee, and informed consent was obtained from all patients before the participation. 

### 2.2. Measurement of Carotid Artery Intima-Media Thickness, Ankle-Brachial Index, and Cardioankle Vascular Index

CA-IMT was measured by high-resolution real-time B mode ultrasonography (GE Logic, Saitama, Japan), as described previously [15]. CA-IMT was defined as the distance between the leading edge of the first echogenic line (lumen-intima interface) and the second echogenic line (media-adventitia interface) of the far wall. Four measurements from both sites were averaged to obtain the mean CA-IMT.

CAVI was measured before the start of dialysis therapy using the VaSera VS-1000 vascular screening system (Fukuda Denshi Co. Ltd., Tokyo, Japan) with the patients resting in a supine position. The principal underlying CAVI has been described previously [13]. Cuffs were wrapped around both ankles and upper arms, which were not used for blood access. Electrocardiographic electrodes were attached to the upper arms, and a microphone was placed on the sternal angle for phonocardiography. After resting for 10 minutes, the examinations were performed. All measurements were automatically calculated using the VaSera VS-1000. Then, blood pressure was measured and the ABI was calculated.

### 2.3. Biochemical Analysis

Blood samples for biochemical parameters were drawn in the morning after overnight fasting. Plasma glucose and serum lipids (cholesterol, high-density lipoprotein- (HDL-) cholesterol, and triglyceride), as well as albumin levels, were measured using standard laboratory methods in the hospital. Hemoglobin A1c (HbA1c) was measured by routine HPLC and latex agglutination immunoassay, which was standardized according to values set by the Japan Diabetes Society [16]. Levels of C-reactive protein (CRP) were measured by nephelometry, a latex particle-enhanced immunoassay (N Latex CRP II; Dade Behring, Tokyo, Japan).

### 2.4. Statistical Analysis

 Statistical analysis was performed using SPSS 20 for Windows. Data were expressed as mean ± standard deviation (SD), median (25th percentiles, 75th percentiles), or percentage. Differences between groups were determined by a test for categorical variables or unpaired *t*-test for continuous variables. Spearman's analysis was applied to examine the relationship between CA-IMT and other clinical parameters. Multiple linear regression analysis was used to determine the contribution of various factors to CA-IMT. A *P* value <0.05 was considered statistically significant.

## 3. Results and Discussion

Clinical and biochemical characteristics of the patients according to diabetic status are shown in Table 1. Compared with patients without diabetes, diabetic patients were found to be older (*P* < 0.001), have a shorter duration of HD (*P* < 0.001), a higher prevalence of CV disease (*P* < 0.05), lower HDL-cholesterol (*P* = 0.009), and higher CRP levels (*P* = 0.02). Systolic blood pressure (SBP) tended to be higher in diabetic patients (*P* = 0.06). Also, by design, the levels of glycemic parameters, such as fasting plasma glucose (FPG) and hemoglobin A1c (HbA1c), in diabetic patients were significantly higher than in those patients without diabetes (*P* < 0.001). CV disease was defined as composite of myocardial infarction, stroke, hospitalization of cardiac heart failure or unstable angina, and coronary or peripheral revascularization.

The proportion of patients with an ABI value of less than 0.95 (0.90) or CAVI value of more than 9 in HD patients with diabetes was also significantly higher than in those without diabetes (Table 1). In this study, the proportion of patients with an ABI value of less than 0.90 was 19.3% (data not shown), which was close to that (17.0%) in the large cohort study of HD patients conducted by Kitahara et al. [12], suggesting that a considerable number of HD patients have the potential to be PAOD.

Regarding the arteriosclerotic parameters, diabetic patients had significantly higher CA-IMT and CAVI values and a lower ABI compared with those without diabetes, as shown in Table 2, although the ABI did not differ between patients with or without diabetes in those patients with an ABI index of more than 0.95. Recently, Chen et al. demonstrated that an ABI value of less than 0.90 was associated with age, pulse pressure, and levels of hematocrit and serum albumin, but not with the presence of diabetes [17]. On the other hand, CAVI in diabetic patients was significantly higher than that in nondiabetic patients, even after excluding patients with ABI of less than 0.95. Collectively, these results suggest that CAVI might be a better index to evaluate arteriosclerosis in HD patients with diabetes, and that ABI might be profoundly affected by many factors other than diabetes. However, in terms of prevalence of CV disease, CAVI did not differ between patients with or without prevalence of CV disease although the patients with prevalence of CV disease have a lower ABI compared with those without prevalence of CV disease (Table 3). A prospective long-term follow-up study is needed to clarify which indices are more important to predict clinical outcome such as new-onset CV disease and death.

In univariate analysis as shown in Table 4, CA-IMT was found to be significantly associated with older age, decreased serum albumin, increased CRP, and increases of glycemic parameters (FPG and HbA1c). Both arteriosclerotic indices (CAVI and ABI) showed a significant correlation with CA-IMT. However, ABI was more closely correlated with CA-IMT than CAVI. These significant arteriosclerotic indices correlated with CA-IMT did not change even after excluding patients with an ABI of less than 0.95, although CAVI was more closely correlated with CA-IMT than ABI at this time. Only in patients with an ABI value of less than 0.95, the correlation between CA-IMT and ABI was still at a borderline level, but that between CA-IMT and CAVI was not.

Furthermore, in only patients with diabetes, CA-IMT was observed to be significantly only associated with age and ABI. On the other hand, in patients without diabetes CA-IMT was significantly associated with serum albumin and CAVI in addition to age and ABI. These results also might reflect that CAVI does not accurately show its value in HD patients with diabetes due to low level of ABI compared to patients without diabetes.

Both baPWV and ABI are noninvasive methods for predicting mortality in both the general population and in HD patients [7, 11, 12, 18]. It has been reported that CAVI is associated with carotid artery arteriosclerosis in patients with hypertension and coronary artery diseases and is also superior to baPWV in terms of its relationship with carotid and coronary artery arteriosclerosis [19–21]. However, little is known about the relationship between CAVI and CA-IMT in HD patients. Recently, Ueyama et al. [22] reported a positive association between maximum CA-IMT and CAVI in HD patients. However, they failed to demonstrate this association in a multiple regression analysis. We also performed a multivariate analysis in order to clarify the independence of the associations of arteriosclerotic indices, which was significant in the Spearman analysis for CA-IMT (Table 5). In addition to age and HbA1c, both arteriosclerotic indices were independently associated with CA-IMT, although serum albumin and CRP were not retained. Since an ABI value of less than 0.95 has been reported to be the cut-off value for diminished pulse wave velocity (PWV) accuracy, such patients were excluded from the analysis and repeated analysis. The variables related to CA-IMT did not change, but CAVI was more closely correlated with CA-IMT than before, and the correlation was almost equal to ABI. In this study, both CAVI and ABI were independently associated with CA-IMT, and almost equally related to CA-IMT in patients with an ABI value of more than 0.95. The reason for the discrepancy is thought to be related to the selection of study patients. Their study excluded patients with ABI of less than 0.90 [22], whereas we selected 0.95 as a cut-off value of ABI. When we use a cut-off value of ABI close to 0.90, ABI became a much better index than CAVI for predicting IMT, but CAVI is still significant determinant. Next, separate analysis was performed for patients with and without diabetes to clarify whether both indices were associated with independently with CA-IMT. As a result, only age and ABI, but not CAVI were determinants for CA-IMT in both cases (data not shown).

## 4. Conclusion

The results led to the following conclusions: (1) CA-IMT in HD patients with T2D or prevalence of CV disease was significantly higher than those without T2D or prevalence of CV disease, respectively, and (2) it appears that ABI is strongly associated with CA-IMT compared to CAVI in HD patients. Because a considerable number of HD patients have a low ABI value, the application of CAVI might be restricted in HD patients.

## Figures and Tables

**Table 1:** Comparison of clinical and biochemical characteristics between hemodialysis patients with or without type 2 diabetes.

Variables	Patients with type 2 diabetes	Patients without type 2 diabetes	*P*
	(*n* = 132)	(*n* = 132)	
Age (yr)	63 ± 10	58 ± 13	<0.001
Male (%)	54.5%	53.0%	0.90
Duration of hemodialysis therapy (yr)	4.8 ± 6.5	7.2 ± 6.5	<0.001
BMI (kg/m^2^)	21.5 ± 3.6	21.1 ± 2.9	0.31
SBP (mmHg)	145 ± 20	141 ± 18	0.06
DBP (mmHg)	77 ± 13	75 ± 10	0.15
Cholesterol (mg/dL)	153 ± 38	159 ± 38	0.16
Triglyceride (mg/dL)	122 ± 68	116 ± 76	0.51
HDL-cholesterol (mg/dL)	46 ± 14	51 ± 16	0.009
Non-HDL-cholesterol (mg/dL)	107 ± 37	109 ± 34	0.72
Serum albumin (mg/dL)	3.6 ± 0.3	3.7 ± 0.3	0.10
FPG (mg/dL)	110 ± 34	79 ± 11	<0.001
HbA1c (%)	5.9 ± 1.0	4.9 ± 0.4	<0.001
CRP (mg/dL)	0.10 (0.08, 0.18)	0.08 (0.06, 0.10)	0.02
History of CV disease (%)	38.9	21.1	0.04
CAVI > 9 (%)	64.3	44.7	0.002
ABI < 0.95 (0.9) (%)	36.4 (25.8)	21.2 (12.9)	0.01 (0.01)

Data are expressed as mean ± SD, median (25th percentiles, 75th percentiles), or percent.

BMI: body mass index; SBP: systolic blood pressure; DBP: diastolic blood pressure; HDL: high-density lipoprotein; FPG: fasting plasma glucose; HbA1c: hemoglobin A1c; CRP: C-reactive protein; CV: cardiovascular, CV; CAVI: cardioankle vascular index; ABI: ankle-brachial index.

**Table 2:** Comparison of arteriosclerosis markers between hemodialysis patients with or without type 2 diabetes according to ABI levels.

	All patients			Patients (ABI ≥ 0.95)		
Variables	Patients with type 2 diabetes	Patients without type 2 diabetes	*P*	Patients with type 2 diabetes	Patients without type 2 diabetes	*P*
	(*n* = 132)	(*n* = 132)		(*n* = 84)	(*n* = 104)	
CA-IMT	0.98 (0.84, 1.18)	0.73 (0.53, 0.92)	<0.001	0.93 (0.80, 1.09)	0.64 (0.50, 0.85)	<0.001
CAVI	9.5 (8.6, 10.3)	8.8 (7.7, 9.6)	<0.001	9.7 (8.6, 10.5)	8.8 (7.8, 9.6)	<0.001
ABI	0.99 (0.89, 1.12)	1.05 (0.97, 1.14)	0.002	1.10 (1.00, 1.14)	1.10 (1.02, 1.17)	0.27

Data are expressed as median (25th percentiles, 75th percentiles).

Abbreviations used in this table are the same as in Table 1.

**Table 3:** Comparison of arteriosclerosis markers between hemodialysis patients with or without prevalence of cardiovascular disease.

Variables	Patients with prevalence of CV disease	Patients without prevalence of CV disease	*P*
	(*n* = 60)	(*n* = 204)	
CA-IMT	0.95 (0.74, 1.18)	0.85 (0.65, 1.05)	0.01
CAVI	9.2 (8.1, 9.8)	9.1 (8.1, 9.9)	0.59
ABI	0.96 (0.85, 1.12)	0.85 (0.65, 1.05)	0.001

Data are expressed as median (25th percentiles, 75th percentiles).

Abbreviations used in this table are the same as in Table 1.

**Table 4:** Spearman's correlation of CA-IMT with clinical markers in all patients, patients with and without diabetes and ABI more or less than 0.95.

Variables	CA-IMT									
	All patients (*n* = 264)		Patients with type 2 diabetes (*n* = 132)		Patients without type 2 diabetes (*n* = 132)		Patients (ABI ≥ 0.95) (*n* = 188)		Patients (ABI < 0.95) (*n* = 76)	
	*r*	*P*	*r*	*P*	*r*	*P*	*r*	*P*	*r*	*P*
Age	0.56	<0.001	0.37	<0.001	0.6700	<0.001	0.56	<0.001	0.35	0.002
Serum albumin	−0.17	0.01	−0.01	0.94	−0.26	0.002	−0.22	0.003	−0.01	0.91
FPG	0.31	<0.001	0.01	0.28	0.11	0.20	0.29	<0.001	0.1	0.40
HbA1c	0.29	<0.001	0.02	0.82	0.03	0.74	0.31	<0.001	0.06	0.64
CRP	0.21	0.001	0.05	0.56	0.13	0.14	0.25	0.001	−0.01	0.93
CAVI	0.25	<0.001	0.10	0.28	0.22	0.01	0.39	<0.001	0.01	0.91
ABI	−0.45	<0.001	−0.35	<0.001	−0.46	<0.001	−0.23	0.002	−0.22	0.05

Abbreviations used in this table are the same as in Table 1.

**Table 5:** Multivariate regression analysis of clinical variables associated with CA-IMT in all patients, patients with ABI ≥ 0.95, and patients with ABI ≥ 0.9.

	CA-IMT								
Variables	All patients (*n* = 264)			Patients (ABI ≥ 0.95) (*n* = 188)			Patients (ABI ≥ 0.9) (*n* = 213)		
	*β*	*t*	*P*	*β*	*t*	*P*	*β*	*t*	*P*
Age	0.45	9.10	<0.001	0.46	7.74	<0.001	0.45	7.81	<0.001
HbA1c	0.21	4.42	<0.001	0.21	3.71	<0.001	0.21	3.90	<0.001
CAVI	0.12	2.51	0.01	0.18	2.98	0.003	0.13	2.29	0.02
ABI	−0.27	−5.33	<0.001	−0.18	−3.17	0.002	−0.20	−3.57	<0.001

*R* ^2^	0.46			0.42			0.42		

Abbreviations used in this table are the same as in Table 1.
